# EIF3B stabilizes PCNA by counteracting SYVN1-mediated ubiquitination to serve as a promotor in cholangiocarcinoma

**DOI:** 10.18632/aging.205759

**Published:** 2024-04-29

**Authors:** Ranglang Huang, Wanpin Nie, Liangliang Mi, Chenjiao Yao, Haixia Zhu

**Affiliations:** 1 Department of Hepatobiliary and Pancreatic Surgery, The Third Xiangya Hospital of the Central South University, Changsha 400013, Hunan, P.R. China; 2 Department of General Medicine, Third Xiangya Hospital, Central South University, Changsha 400013, Hunan, P.R. China

**Keywords:** cholangiocarcinoma, EIF3B, PCNA, P21

## Abstract

Cholangiocarcinoma, a prevalent hepatic malignancy, exhibits a progressively rising incidence. While Eukaryotic translation initiation factor 3 subunit B (EIF3B) has been implicated in the occurrence and development of various cancers, its specific roles in cholangiocarcinoma remain unexplored. Immunohistochemical (IHC) analysis was employed to detect EIF3B/PCNA expression in cholangiocarcinoma. Cells were manipulated using short hairpin RNA (shRNA)-mediated lentiviruses or overexpression plasmids. Statistical significance was assessed using the Student’s t-test and one-way ANOVA, with P < 0.05 considered statistically significant. EIF3B exhibited robust expression in cholangiocarcinoma, demonstrating a significant correlation with the pathological grade of cholangiocarcinoma patients. Furthermore, modulation of EIF3B expression, either depletion or elevation, demonstrated the ability to inhibit or enhance cholangiocarcinoma cell survival and migration *in vitro*. Mechanistically, we identified Proliferating Cell Nuclear Antigen (PCNA) as a downstream gene of EIF3B, driving cholangiocarcinoma. EIF3B stabilized PCNA by inhibiting PCNA ubiquitination, a process mediated by E3 ligase SYVN1. Similar to EIF3B, PCNA levels were also abundant in cholangiocarcinoma, and knocking down PCNA impeded cholangiocarcinoma development. Intriguingly, silencing PCNA attenuated the promotion induced by EIF3B overexpression. Furthermore, the elevated P21 protein level in shEIF3B RBE cells was partially attenuated after UC2288 (P21 signaling pathway inhibitor) treatment. Our findings underscored the potential of EIF3B as a therapeutic target for cholangiocarcinoma. Unraveling its functions holds promise for the development of more specific and effective targeted therapy strategies.

## INTRODUCTION

Cholangiocarcinoma ranks as the second most prevalent hepatic malignancy following hepatocellular carcinoma [1]. Its global incidence has steadily risen over the past four decades [2–4]. The classification of cholangiocarcinoma into three subtypes based on its location introduces unique aspects of epidemiology, biology, prognosis, and clinical management for each subtype [5]. Surgical resection remains the preferred treatment approach [6–8], with chemotherapy reserved for cases where the disease is advanced or unresectable [9, 10]. Furthermore, the identification of promising molecular targets, guided by the genetic patterns of each subtype, has significantly enhanced survival outcomes [11, 12]. In the context of this study, our aim was to uncover additional therapeutic targets for cholangiocarcinoma, contributing to the ongoing efforts to broaden the treatment options and improve the overall management of this challenging malignancy.

The process of translation relies on the involvement of multiple initiation factors [13], among which the eukaryotic translation initiation factor (EIF) holds a crucial role in the formation of initiation complexes [14]. The EIF family comprises 13 members, collectively contributing to the entire translation initiation process [15]. Additionally, studies indicate the significant involvement of the EIF3 complex, the largest and most intricate factor within the EIF family, in tumorigenesis [16]. Various members of the EIF3 family, such as EIF3A and EIF3D, have been identified as overexpressed in diverse cancer types, including lung, breast [17], cervix [18], stomach [19] and esophagus [20]. A prior study demonstrated that EIF3B knockdown suppresses U87 cell proliferation, induces G0/G1-phase arrest, and accelerates cell apoptosis [21]. Additionally, EIF3D has been reported to play an oncogenic role in glioma [22], colon cancer [23], and non-small cell lung cancer [24]. In this study, our focus centers on EIF3B, a scaffold protein within the EIF3 complex. Previous research has highlighted EIF3B’s involvement in translation regulation [15], cell growth, and its crucial role in tumorigenesis [25]. Elevated levels of EIF3B have been linked to poor prognosis in clear cell renal cell carcinoma [26], esophageal squamous cell carcinoma [27], gastric cancer [28], and bladder cancer [29]. However, limited information is available regarding its relevance to cholangiocarcinoma.

The presented study revealed a significant upregulation of EIF3B in cholangiocarcinoma, demonstrating a strong correlation with the pathological grade of patients. Depletion of EIF3B was shown to impede the progression of cholangiocarcinoma both *in vitro* and *in vivo*. The tumor-promoting impact of EIF3B was found to be mediated through PCNA, and the inhibition of PCNA not only suppressed the development of cholangiocarcinoma but also reversed the promotional effects induced by EIF3B overexpression. Mechanistically, the silencing of EIF3B in RBE cells led to an increase in P21 protein levels, and this effect was attenuated following treatment with UC2288, a P21 signaling pathway inhibitor. In summary, these findings indicated the potential of EIF3B as a promising therapeutic target for cholangiocarcinoma.

## RESULTS

### Increased EIF3B expression in cholangiocarcinoma predicted poor patient overall survival (OS)

**Figure 1:**
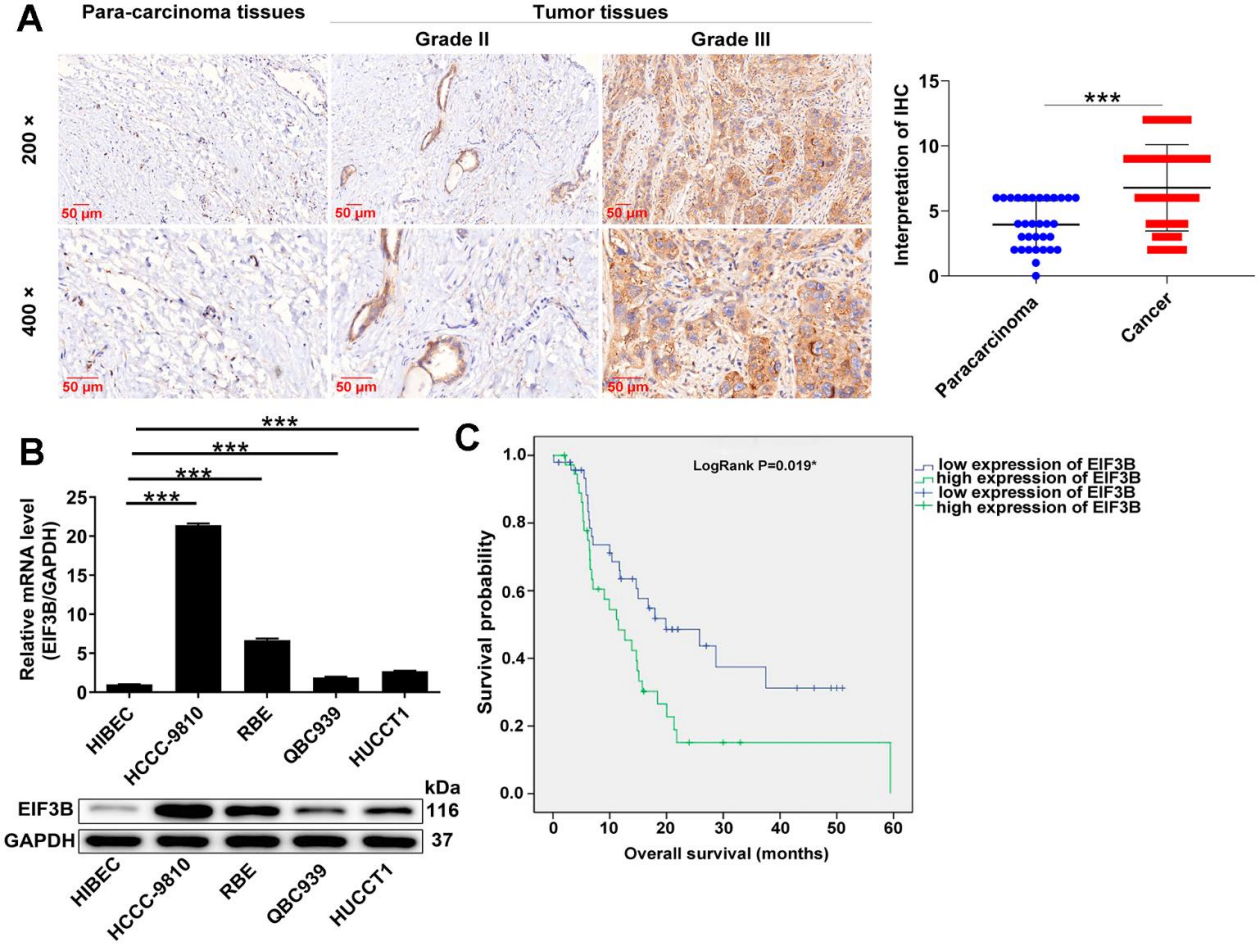
**EIF3B was abundantly expressed in cholangiocarcinoma tissues and cells.** (**A**) Typical images and quantitative data illustrations of EIF3B immunohistochemical staining in cholangiocarcinoma tissues and para-carcinoma tissues. Scale bar: 50 μm. Magnification times: 200 ×, 400 ×. (**B**) EIF3B mRNA and protein levels in cholangiocarcinoma cell lines (HCCC-9810, RBE, HUCCT1, QBC939) and HIBEC cell lines. The experiments were in triplicate. (**C**) Kaplan-Meier survival analysis was performed to reveal the relationship between EIF3B expression and prognosis of cholangiocarcinoma patients. Results were presented as mean ± SD. *** *P* < 0.001.

EIF3B expression levels in cholangiocarcinoma tissues and cells were assessed. IHC analysis revealed that EIF3B was abundant in cholangiocarcinoma tissues but rarely present in para-carcinoma tissues (*P* < 0.001, Figure 1A and Table 1). Moreover, four cholangiocarcinoma cell lines used here, namely HCCC-9810, RBE, HUCCT1 and QBC939, exhibited higher EIF3B mRNA and protein levels compared with the non-cholangiocarcinoma cell line HIBEC, especially in HCCC-9810 and RBE (Figure 1B), implying the potential significance of EIF3B in promoting cholangiocarcinoma. We further evaluated the correlation between EIF3B with clinicopathological characteristics of patients with cholangiocarcinoma. Combining Mann-Whitney U analysis and Spearman’s rank correlation analysis, we found that elevated EIF3B was significantly associated with pathological grade (Tables 2, 3). In addition, Kaplan-Meier survival analysis revealed that increased EIF3B expression predicted poor overall survival (OS) for patients with cholangiocarcinoma (Figure 1C). On this account, EIF3B might act as a potential promotor in the development cholangiocarcinoma development.

**Table 1:** Expression patterns of EIF3B in cholangiocarcinoma tissues and normal tissues revealed in immunohistochemistry analysis.

**EIF3B expression**	**Tumor tissue**		**Para-carcinoma tissue**		***P*-value**
	**Cases**	**Percentage**	**Cases**	**Percentage**	
Low	50	55.6%	31	100%	< 0.001
High	40	44.4%	-	-	

**Table 2:** Relationship between EIF3B expression and tumor characteristics in patients with cholangiocarcinoma.

**Features**	**No. of patients**	**EIF3B expression**		***P*-value**
		**low**	**high**	
All patients	90	50	40	
Age (years)				0.673
<58	45	26	19	
≥ 58	45	24	21	
Gender				0.778
Male	48	26	22	
Female	42	24	18	
Grade				0.002
I	5	5	0	
II	67	40	27	
III	18	5	13	
lymphatic metastasis (N)				0.551
N0	60	32	28	
N1	30	18	12	
Tumor infiltrate				0.861
T1	7	2	5	
T2	60	37	23	
T3	19	8	11	
T4	3	2	1	
Tumor size				0.171
≤ 3cm	50	31	19	
>3cm	40	19	21	
Lymph node positive				0.439
≤ 0	60	32	28	
>0	29	18	11	
Stage				0.510
1	8	1	7	
2	40	26	14	
3	28	15	13	
4	14	8	6	

**Table 3:** Relationship between EIF3B expression and tumor characteristics in patients with cholangiocarcinoma.

		**EIF3B**
Grade	Spearman correlation	0.331
	Signification (double-tailed)	0.001
	N	90

### EIF3B depletion suppressed cholangiocarcinoma cell survival and migration *in vitro*

**Figure 2:**
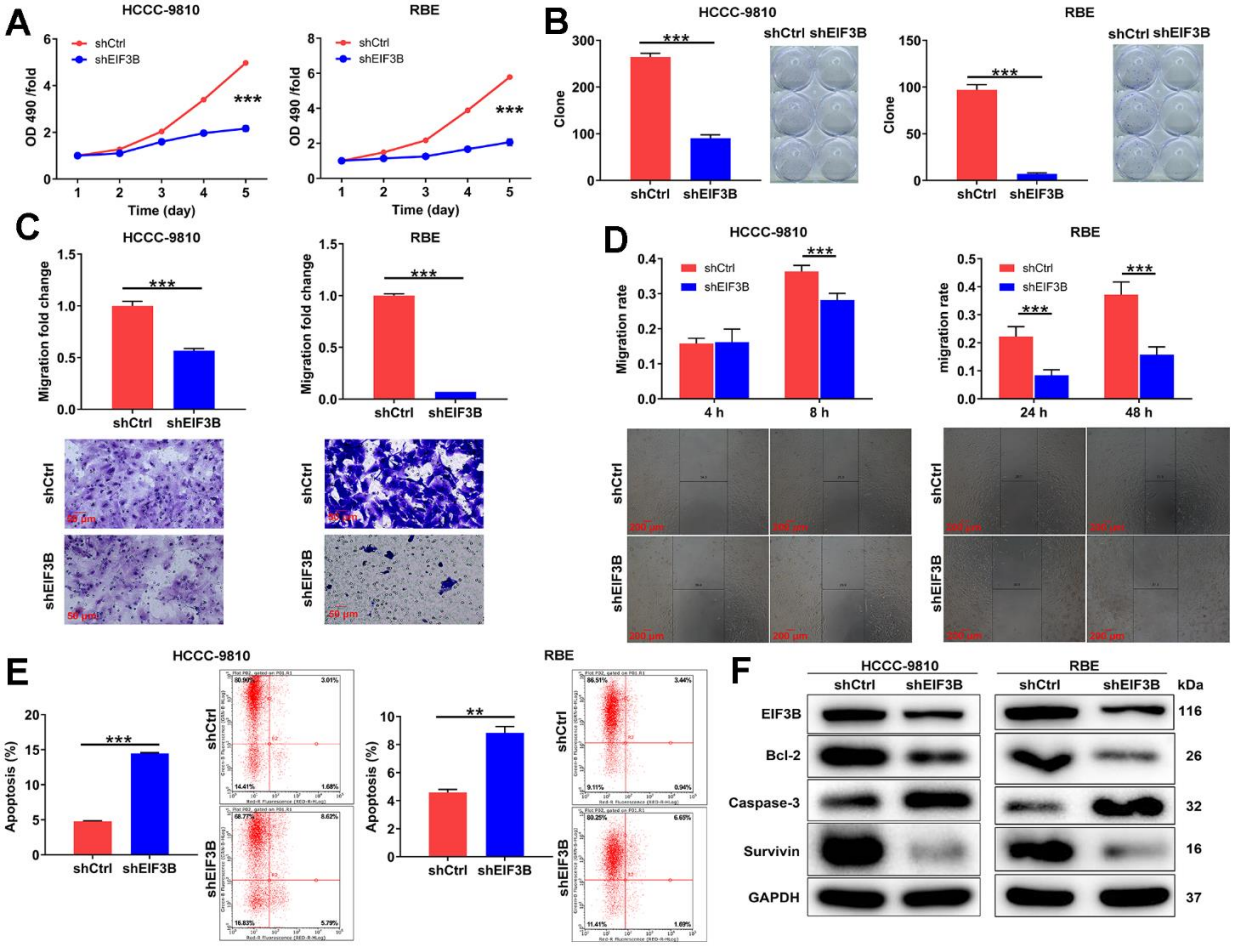
**EIF3B depletion suppressed cholangiocarcinoma development *in vitro*.** (**A**) The cell proliferation abilities of HCCC-9810 and RBE cells after being transfected shEIF3B or shCtrl were assessed by MTT assay. (**B**) After being transfected shEIF3B or shCtrl, the capacities of HCCC-9810 and RBE cells to form colony were detected. (**C**, **D**) After being transfected shEIF3B or shCtrl, the changes of cell migration of HCCC-9810 and RBE cells were evaluated by transwell assay (**C**) and wound-healing assay (**D**). Scale bar: 50 μm for transwell assay and 200 μm for wound-healing assay. Magnification times: 200 × for transwell assay and 50 × for wound-healing assay. (**E**) The effects of EIF3B knockdown on cell apoptosis of HCCC-9810 and RBE cells were examined by flow cytometry. (**F**) The levels of apoptosis-related proteins in HCCC-9810 and RBE cells transfected with shEIF3B and shCtrl were measured by western blot. All the experiments were in triplicate. Results were presented as mean ± SD. ** *P* < 0.01, *** *P* < 0.001.

In this section, our aim was to elucidate the roles of EIF3B in cholangiocarcinoma development. Consequently, we knocked down EIF3B in HCCC-9810 and RBE cells, achieving transfection efficiencies exceeding 80%, and observed a substantial decrease in EIF3B mRNA and protein levels (Supplementary Figure 1). Subsequently, we assessed the impact of EIF3B depletion on cholangiocarcinoma cell phenotypes. As expected, the cells in shEIF3B groups exhibited a slower proliferation rate compared to the shCtrl groups (*P* < 0.001, Figure 2A). Additionally, Figure 2B illustrated that the capacity for colony formation was markedly suppressed upon knocking down EIF3B (*P* < 0.001). Furthermore, cell migration levels were evaluated through wound-healing assay and transwell assay, indicating that both shEIF3B-transfected cells exhibited lower migration levels than their corresponding shCtrl-transfected cells (*P* < 0.001, Figure 2C, 2D). More importantly, EIF3B depletion significantly enhanced cell apoptosis (*P* < 0.001 for HCCC-9810 cells, *P* < 0.01 for RBE cells, Figure 2E). Notably, the levels of apoptosis-related proteins, including Bcl-2, caspase-3, and Survivin, were assessed through western blot in HCCC-9810 and RBE cells with shCtrl and shEIF3B. As anticipated, the results demonstrated a downregulation of Bcl-2 and Survivin, along with an upregulation of caspase-3 upon EIF3B knockdown (Figure 2F), implying that EIF3B might be involved in cholangiocarcinoma cell apoptosis through regulating these proteins. Taken together, we concluded that EIF3B promoted cholangiocarcinoma cell growth and migration, while suppressing cell apoptosis *in vitro*.

### PCNA was identified as a downstream gene of EIF3B regulating cholangiocarcinoma

**Figure 3:**
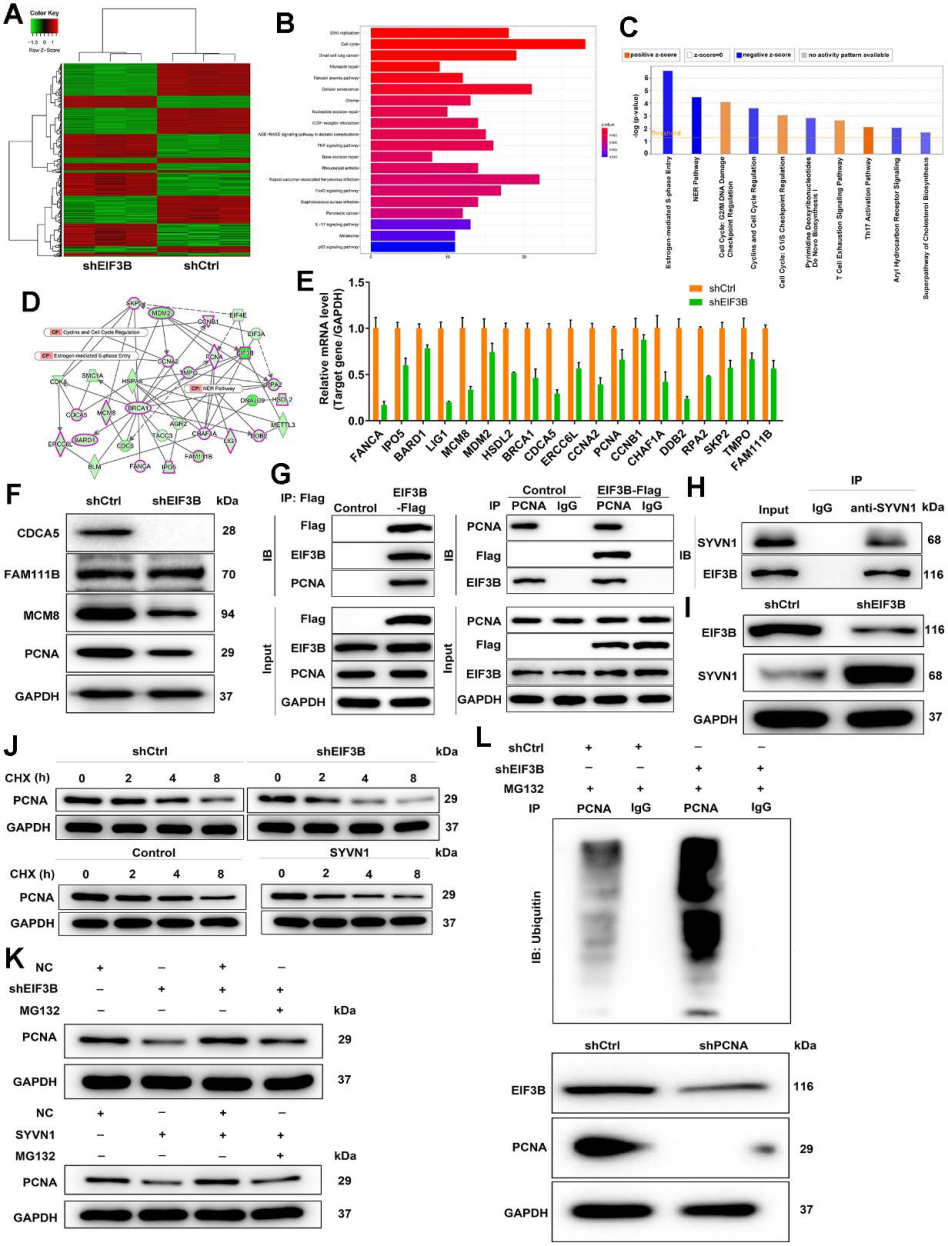
**Exploration and verification of underlying mechanism of EIF3B regulating cholangiocarcinoma.** (**A**) Gene expression profile from PrimeView Human Gene Expression Array analysis in shCtrl/shEIF3B-transfected HCCC-9810 cells. (**B**, **C**) The enrichment of the differentially expressed genes (DEGs) in canonical signaling pathways was analyzed by Kyoto Encyclopedia of Genes and Genomes (KEGG) (**B**) and IPA (**C**). (**D**) Interaction network based on IPA illustrating the impact of EIF3B on key genes. (**E**, **F**) qRT-PCR (E) and western blot (**F**) analysis were used to detect the levels of candidate DEGs in HCCC-9810 cells with shEIF3B or shCtrl. (**G**, **H**) Co-immunoprecipitation (Co-IP) experiment demonstrating the exogenous interaction between EIF3B and PCNA in 293T cells (**G**), and the endogenous interaction between SYVN1 and EIF3B in HCCC-9810 cells (**H**). (**I**) Analysis of SYVN1 protein levels in HCCC-9810 cells upon EIF3B depletion. (**J**) Investigation of PCNA protein stability in HCCC-9810 cells with EIF3B knockdown or SYVN1 overexpression following 0.2 mg/mL CHX treatment for indicated times. (**K**) Impact of the proteasome inhibitor MG-132 on the degradation of PCNA protein following EIF3B knockdown or SYVN1 overexpression. (**L**) The lysates of HCCC-9810 cells with EIF3B knockdown were immunoprecipitated, and western blot was performed to examine the ubiquitination of PCNA. All the experiments were in triplicate.

To investigate the mechanism behind EIF3B knockdown blocking cholangiocarcinoma progression, we conducted a PrimeView Human Gene Expression Array on shCtrl/shEIF3B-transfected HCCC-9810 cells, identifying 402 upregulated and 442 downregulated genes in the shEIF3B group (Figure 3A). Subsequent pathway enrichment analyses underscored the enrichment of the cell cycle pathway, specifically highlighting the NER Pathway, Estrogen-mediated S-phase Entry, and Cyclins and Cell Cycle Regulation (Figure 3B, 3C). The interaction network based on IPA revealed that EIF3B influenced BARD1, BRCA1, CCNA2, CCNB1, CDCA5, CHAF1A, DDB2, ERCC6L, FAM111B, FANCA, HSDL2, IPO5, LIG1, MCM8, MDM2, PCNA, RPA2, SKP2 and TMPO in these pathways (Figure 3D). To further validate our findings, we performed qPCR on these key genes, observing a consistent decrease in these genes in response to EIF3B depletion (Figure 3E). Additionally, a differential expression analysis of these genes based on cholangiocarcinoma and normal tissues samples from the TCGA database revealed significant upregulation of CDCA5, FAM111B, MCM8 and PCNA in cholangiocarcinoma, supporting their relevance in this context (Supplementary Figure 2). Subsequent western blot experiments corroborated a noticeable downregulation in CDCA5, MCM8, and PCNA in response to EIF3B depletion (Figure 3F). While CDCA5 appeared to be more prominently regulated by EIF3B, we selected PCNA as a downstream target due to its well-established role as cell cycle marker. Also, expression of PCNA is a universal feature in cholangiocarcinoma [30]. Here, we also found that PCNA was abundantly expressed in cholangiocarcinoma tissues and cells (Supplementary Figure 3A, 3B). More importantly, there was a protein interaction between EIF3B and PCNA (Figure 3G). Next, we aimed to explore how EIF3B regulates PCNA expression. Through analyzing the ubibrowser website (http://ubibrowser.ncpsb.org.cn/ubibrowser/strict/networkview/networkview/name/P12004/jobId/ubibrowse-I2021-12-01-21544-1638350113), we identified SYVN1 as an E3 ubiquitin ligase of PCNA. Co-IP experiment indicated that SYVN1 could endogenously interact with EIF3B (Figure 3H). Additionally, the depletion of EIF3B resulted in a notable upregulation of SYVN1 protein levels in HCCC-9810 cells (Figure 3I).

We accordingly hypothesized that EIF3B could regulate PCNA expression through SYVN1-mediated PCNA ubiquitination. To verify this hypothesis, we initially examined the protein stability of PCNA in HCCC-9810 cells with EIF3B knockdown or SYVN1 overexpression, and found a significant acceleration in the degradation of PCNA protein under both conditions (Figure 3J and Supplementary Figure 3C). Additionally, treatment with the proteasome inhibitor MG-132 partially attenuated the degradation of PCNA protein following EIF3B knockdown or SYVN1 overexpression (Figure 3K), suggesting a potential role for EIF3B in regulating PCNA through the ubiquitin-proteasome system (UPS). Given that protein ubiquitination is commonly linked with proteasome-mediated degradation, [31, 32], we subsequently evaluated the level of PCNA ubiquitination. As depicted in Figure 3L, downregulation of EIF3B significantly increased the ubiquitination of PCNA, concomitant with a decrease in the protein level of PCNA.

### EIF3B mediated cholangiocarcinoma progression through regulating PCNA

**Figure 4:**
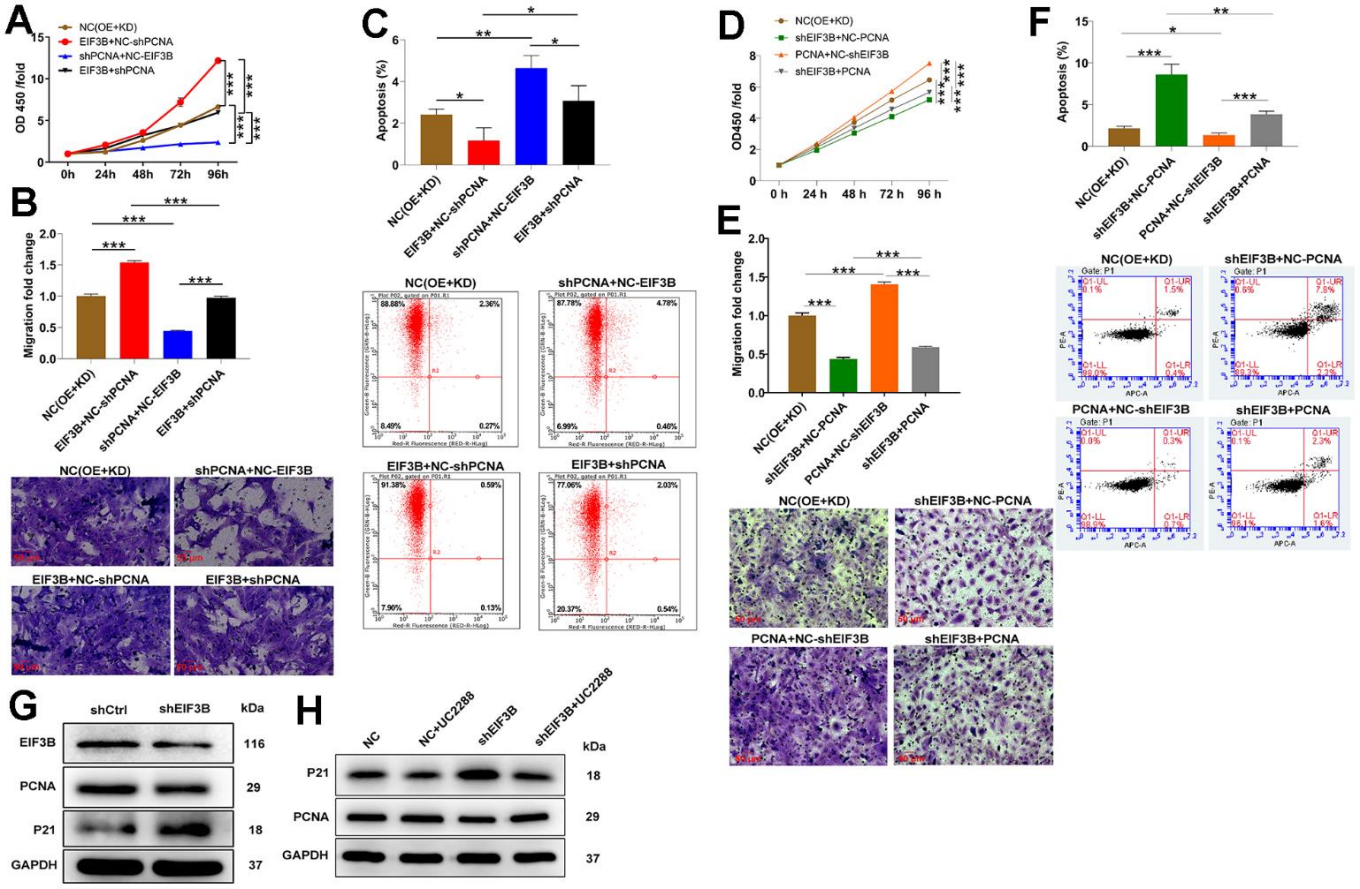
**EIF3B mediated cholangiocarcinoma progression through regulating PCNA.** (**A**–**C**) Validation of the effects of EIF3B overexpression on the inhibitory effects of PCNA knockdown on cholangiocarcinoma progression, including proliferation (**A**), migration (**B**), and apoptosis (**C**), in REB cells. Scale bar: 50 μm for transwell assay. Magnification times: 200 ×. NC (OE+KD): Control, EIF3B+NC-shPCNA: EIF3B overexpression, shPCNA+NC-EIF3B: PCNA downregulation, EIF3B+shPCNA: EIF3B overexpression plus PCNA downregulation. (**D**–**F**) Validation of the effects of EIF3B knockdown on the promoting effects of PCNA overexpression on cholangiocarcinoma progression, including proliferation (**D**), migration (**E**), and apoptosis (**F**), in REB cells. Scale bar: 50 μm for transwell assay. Magnification times: 200 ×. NC (OE+KD): Control, PCNA+NC-shEIF3B: PCNA overexpression, shEIF3B+NC-EIF3B: EIF3B downregulation, PCNA+shEIF3B: PCNA overexpression plus EIF3B downregulation. (**G**) The expression of EIF3B, PCNA and P21 in HCCC-9810 cells transfected with shEIF3B and shCtrl was detected by western blot. (**H**) HCCC-9810 cells transfected with shEIF3B and shCtrl were treated with UC2288, and the levels of PCNA and P21 were detected by western blot. All the experiments were in triplicate. The data are expressed as mean ± SD. * *P* < 0.05, ** *P* < 0.01, *** *P* < 0.001.

To confirm the contribution of PCNA to EIF3B-mediated cholangiocarcinoma progression, we established RBE cell models involving singular overexpression of EIF3B, singular silence of PCNA, and concurrent EIF3B overexpression with PCNA silencing. Transfection efficiency was assessed through qRT-PCR and western blot (Supplementary Figure 3D, 3E). Utilizing the Celigo cell counting assay, we observed that HCCC-9810 cells in the in EIF3B+NC-shPCNA group exhibited accelerated proliferation (*P* < 0.001), while those in the shPCNA+NC-EIF3B group displayed the opposite trend (*P* < 0.001). Importantly, the enhanced cell proliferation induced by EIF3B overexpression was mitigated upon PCNA knockdown (*P* < 0.01, Figure 4A). Moreover, EIF3B overexpression not only stimulated cell proliferation but also induced cell migration, and both effects were reversed by silencing PCNA (Figure 4B). Regarding apoptosis, there was a noticeable trend towards decreased cell apoptosis after EIF3B overexpression (*P* < 0.01), which was counteracted by PCNA knockdown (*P* < 0.001, Figure 4C). To further validate whether PCNA could reverse the inhibitory effects of EIF3B knockdown on cholangiocarcinoma, we conducted separate transfections of a PCNA overexpression plasmid and an EIF3B knockdown plasmid, as well as simultaneous transfections, in REB cells. Transfection efficiency was assessed through qRT-PCR and western blot (Supplementary Figure 3D, 3E). Remarkably, PCNA overexpression effectively rescued the tumor-inhibitory effects induced by EIF3B knockdown, resulting in heightened proliferation and migration, alongside the inhibition of apoptosis (Figure 4D–4F). Overall, our findings demonstrated that EIF3B was involved in cholangiocarcinoma development through targeting PCNA.

### EIF3B promoted cholangiocarcinoma development via inhibiting P21 pathway

On the other hand, considering the enrichment of most genes in the cell cycle pathway and the recognized functional roles of P21 in cell cycle regulation, we postulated the involvement of theP21 pathway in EIF3B-mediated cholangiocarcinoma. Consequently, we assessed the P21 protein levels in HCCC-9810 cells following the knockdown of EIF3B, revealing a concurrent upregulation of P21 (Figure 4G). To further investigate this connection, we treated shEIF3B HCCC-9810 cells with the P21 signaling pathway inhibitor UC2288, resulting in a diminished P21 level in the shEIF3B+UC2288 group compared to the shEIF3B group. Intriguingly, the previously attenuated PCNA expression was heightened after UC2288 treatment (Figure 4H). These data showed that EIF3B promoted cholangiocarcinoma development via inhibiting the P21 pathway.

### EIF3B depletion suppressed cholangiocarcinoma tumor growth *in vivo*

**Figure 5:**
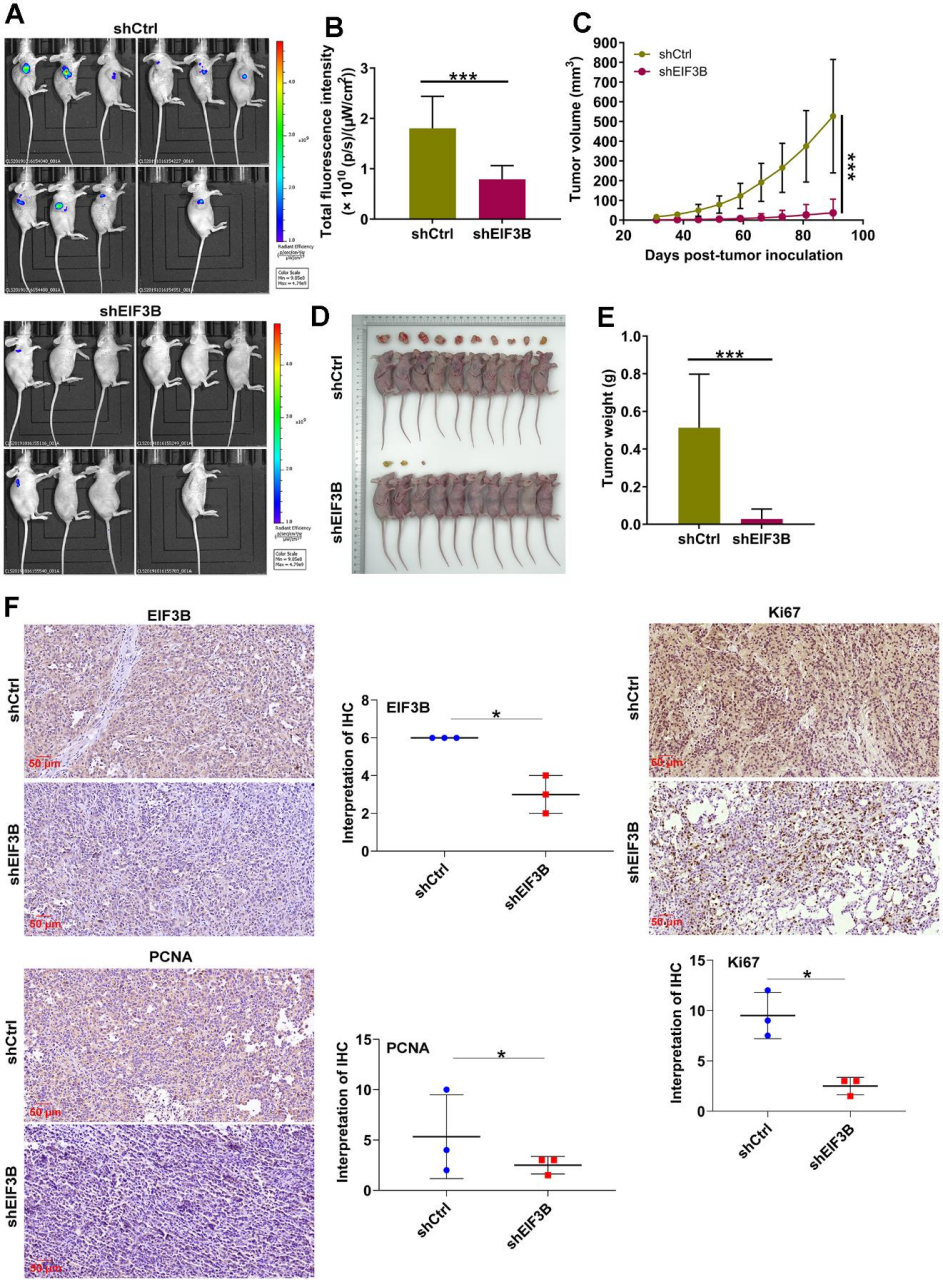
**EIF3B knockdown inhibited cholangiocarcinoma tumor growth *in vivo*.** (**A**) Establishment of animal models by implanting EIF3B-knockdown and control RBE cells into female BALB/c nude mice for investigating the role of EIF3B in cholangiocarcinoma tumor growth. (**B**) The fluorescence intensity in shCtrl and shEIF3B groups was obtained through injecting D-Luciferase. (**C**–**E**) Evaluation of tumor growth parameters in the animal models, including tumor volume (**C**), size (**D**), and weight (**E**). (**F**) Typical images and quantitative data illustrations of EIF3B, PCNA and Ki67 immunohistochemical staining in mice tumor tissues. Scale bar: 50 μm. Magnification times: 200 ×. Results were presented as mean ± SD. * *P* < 0.05, *** *P* < 0.001.

To ascertain the crucial role of EIF3B in cholangiocarcinoma tumor growth, animal models were established by implanting EIF3B-knockdown RBE cells into female BALB/c nude mice (Figure 5A). The results revealed that the shEIF3B group exhibited significantly lower fluorescence intensity, smaller tumor size, as well as reduced weight and volume compared to the shCtrl group (Figure 5B–5E). After 90 days, tumor tissues were harvested for IHC analysis, demonstrating a marked downregulation of EIF3B, PCNA and Ki-67 levels in the EIF3B knockdown group (Figure 5F). These indicated that EIF3B is essential not only for cell proliferation and migration *in vitro* but also for efficient tumor growth *in vivo*.

## DISCUSSION

Targeted therapy for various cancer types is currently centered around key genetic mutations that activate tumor growth. Several small molecule inhibitors, such as NVP-BGJ398, Erdafitinib, Derazantinib, TAS-120, and Debio 1347, are in the early phases of clinical trials and have shown promise as treatments for cholangiocarcinoma [11, 12, 33]. However, limitations and side effects associated with these drugs have been observed in patients [34], highlighting the necessity for novel and more effective agents for cholangiocarcinoma. EIF3B, which is abnormally expressed in various human cancers and plays a crucial role in tumor growth, has been studied in different cancer types, including glioblastoma [21], bladder cancer [29], esophageal squamous cell carcinoma [27] and prostate cancer [35]. However, its functional role in cholangiocarcinoma is not yet clear.

We initiated our study by examining the role of EIF3B in the development and progression cholangiocarcinoma. Our results showed abundant EIF3B expression in both cholangiocarcinoma tissues and cell lines, exhibiting a correlation with pathological grade. Through the use of shEIF3B-expressing lentivirus, we successfully silenced EIF3B and observed a consequential tumor suppression effect. This downregulation of EIF3B inhibited cell proliferation, migration and enhanced apoptosis, a phenomenon also substantiated by *in vivo* data. To delve into the underlying mechanism of EIF3B’s influence on cholangiocarcinoma, we further investigated its impact on PCNA. Silencing EIF3B resulted in a significant decrease in both PCNA mRNA and protein levels. The validation of the interaction between PCNA and EIF3B indicated that PCNA serves as a downstream factor regulated by EIF3B in the context of cholangiocarcinoma.

PCNA, a well-established marker of the cell cycle, was initially identified by Miyachi et al*.* in 1978, named for its discovery in the nuclei of dividing cells [36]. This protein plays a pivotal role in nucleic acid metabolism, participating in crucial functions such as DNA replication [37], regulation of DNA excision repair [38], and control of the cell cycle [39, 40]. During the S phase, PCNA interacts with the CDK2-cyclin A complex, facilitating the phosphorylation of CDK2 substrates and directing the complex to PCNA-binding DNA replication proteins [39]. Moreover, PCNA is also responsible for tumor growth, serving as a marker for breast cancer cell proliferation and prognosis [41]. In the context of cholangiocarcinoma, elevated PCNA expression designates it as a tumor promoter. Depletion of PCNA impedes cell proliferation and migration while concurrently promoting apoptosis.

The ubiquitin-proteasome system (UPS) is integral to protein quality control and cellular homeostasis [42]. Dysfunctions within the UPS is associated with various diseases, including cancer, where components of the UPS are often mutated or abnormally expressed [43]. In this study, we uncovered an interaction between EIF3B and the E3 ligase SYVN1. This interaction influences SYVN1-mediated ubiquitination of PCNA. Knockdown of PCNA significantly weakened the regulatory functions of EIF3B, resulting in hindered cell proliferation and migration, and encouraged apoptosis. This shed light on a novel regulatory mechanism involving EIF3B, SYVN1, and PCNA in cholangiocarcinoma pathogenesis.

On the flip side, upon silencing EIF3B, we observed an upregulation in P21 protein levels, a key regulator of the cell cycle. P21 is involved in fundamental cellular growth control, stem cell regulation, differentiation, and stress response [44]. Additionally, P21 expression acts as a suppressor of tumor growth [45] and exhibits specific functions during the S-phase, regulating genomic stability through its interaction with PCNA [46]. To investigate this further, we subjected shEIF3B RBE cells to treatment with the P21 signaling pathway inhibitor UC2288. This intervention resulted in a reduction in P21 levels and a concurrent increase in PCNA expression, providing valuable insights into the intricate interplay between EIF3B, P21, and PCNA, and shedding light on potential regulatory mechanisms in cellular processes and their implications for tumorigenesis.

In conclusion, our study elucidated that EIF3B played a pivotal role in promoting cholangiocarcinoma through the regulation of the PCNA and P21 pathways. These findings suggested that EIF3B could serve as a promising target for the treatment of this disease.

## MATERIALS AND METHODS

### Tissue specimens and cell lines

The tissue microarrays utilized in this study were supplied by Xi’an Alenabio Co., Ltd. (Xi’an, China), encompassing 90 cholangiocarcinoma tissues and 31 normal tissues. Individuals with a prior primary malignancy in other organs before diagnosis or those who underwent radiotherapy and chemotherapy were excluded from the study. Human intrahepatic bile duct epithelial cells (HIBEC) and four types of human cholangiocarcinoma cell lines (HCCC-9810, RBE, QBC939, HUCCT1) were acquired from National Infrastructure Cell Line Resource (Beijing, China). HIBEC cells were cultured in MEM (Meilunbio Biological Technology Co., Ltd., China, #MA0217) supplemented with 10% FBS (Junzhe Biotechnology Co., Ltd., #04-001-1ACS), while HUCCT1, HCCC-9810 and RBE cells were cultured in 1640 medium (Meilunbio Biological Technology Co., Ltd., #MA0215) with 10% FBS. QBC939 cells were cultured in H-DMEM (Meilunbio Biological Technology Co., Ltd., #MA0212) with 10% FBS. The cells were incubated in a 37° C incubator containing 5% CO_2_. STR profiling of the cell line was conducted to authenticate it, and mycoplasma contamination was then tested.

### Immunohistochemistry (IHC) staining

The tissue samples underwent xylene immersion and alcohol washing (China National Pharmaceutical Group Co., Ltd., Beijing, China) before undergoing slide repair with 1×EDTA (Beyotime Biotechnology Co., Ltd., Shanghai, China). Subsequently, the slides were blocked with 3% H_2_O_2_ and serum. Incubation with EIF3B, PCNA, or Ki-67 antibodies followed, and the slides were stained with DAB and hematoxylin (Baso Diagnostics Inc., Zhuhai, China), finally being sealed with neutral resin (China National Pharmaceutical Group Co., Ltd., Beijing, China). The IHC images were captured using an optical microscope and meticulously analyzed by three pathologists in a randomized fashion. Positive cell scores were stratified as follows: 1 (1%-24%), 2 (25%-49%), 3 (50%-74%), and 4 (75%-100%). Staining intensity was assessed on a scale of 0 (signalless color), 1 (light yellow), 2 (brown yellow), to 3 (dark brown). The IHC results were determined by multiplying the positive cell score by the staining color intensity score, with a higher score indicating elevated antibody expression. The scoring system was defined as follows: 0 (negative), 1-4 (positive), 5-8 (positive++), 9-12 (positive+++). To categorize high and moderate expression, criteria were established based on the median of IHC scores across all tissue samples. Samples with scores exceeding the median were categorized as high expression, while those with scores below the median were classified as low expression. Antibodies used in this section were listed in Supplementary Table 1.

### Plasmid construction and lentivirus transfection

The corresponding RNAi target sequences for EIF3B and PCNA (EIF3B: GGGAGAGAAATTCAAGCAAAT; PCNA: TACACTAAGGGCCGAAGATAA), along with a control RNAi scramble sequence (TTCTCCGAACGTGTCACGT), were designed by Shanghai Biosciences Co., Ltd. (Shanghai, China). Subsequently, the BR-V-108 vector was loaded with the target sequences, which were then inserted through the restriction sites at both ends and transformed into TOP 10 *E. coli* competent cells (Tiangen, Beijing, China). Additionally, using EIF3B as a template, a primer amplification sequence (F-AGGTCGACTCTAGAGGATCCCGCCACCATGCAGGACGCGGAGAACGTGGCG; R-TCCTTGTAGTCCATACCCTCCTGATTCCCGAGGGGAATGATTTC) was designed for constructing a lentiviral vector for EIF3B overexpression. The concentration of positive recombinant plasmids was determined using the EndoFree maxi plasmid kit (Tiangen, Beijing, China) and a Thermo_Nanodrop 2000 spectrophotometer. To transfect lentivirus into HCCC-9810 and RBE cells, 20 μL of 1×10^8^ TU/mL lentivirus was added to the logarithmically growing cells under ENI.S+Polybrene infected conditions. Subsequently, the cells were cultured in 1640 medium, and the transfection as well as knockdown/overexpression efficiencies were evaluated.

### RNA extraction, cDNA synthesis and qRT-PCR

To extract total RNA, TRIzol reagent (Sigma-Aldrich, St. Louis, MO, USA) was utilized. Subsequent to isolating DNA, cDNA synthesis and qRT-PCR were conducted on cholangiocarcinoma cell lines transfected with indicated lentivirus, employing the Promega M-MLV Kit (Promega Corporation, Madison, WI, USA) and the SYBR Green Mastermixs Kit (Vazyme, Nanjing, China). GAPDH served as an internal normalization control, and the 2^-∆∆Ct^ method was employed to evaluate relative mRNA expression. The primers sequences (5′-3′) were listed in Supplementary Table 2.

### Western blot assay and co-immunoprecipitation (Co-IP)

Proteins were collected from lentivirus-transfected HCCC-9810 and RBE cells and lysed using 1× Lysis Buffer (Cell Signal Technology, Danvers, MA, USA). The BCA Protein Assay Kit (HyClone-Pierce, #23225) was employed to determine protein purity, and 10% SDS-PAGE was utilized for protein segregation. The membranes were incubated with primary and secondary antibodies, washed three times, and color development was performed using the ECL+plusTM western blotting system kit. For the Co-IP assay, proteins were immunoprecipitated with anti-PCNA, anti-SYVN1, or IgG antibodies and subsequently subjected to western blotting with specific antibodies. Details of the antibodies used were showed in Supplementary Table 1.

### Cell proliferation detection

For the MTT assay, cholangiocarcinoma cell lines treated with lentivirus expressing shEIF3B were cultured and incubated with MTT for five days. The optical density (OD) value was measured using a microplate reader (Tecan Infinite, Mannedorf Zurich, Switzerland).

In the CCK-8 detection protocol, RBE cells transfected with lentivirus as specified were treated according to the aforementioned procedure. Subsequently, on the following day, CCK-8 reagent was introduced, and after a 4-h incubation period, the optical density (OD) was measured at 450 nm using a microplate reader. The proliferation curve was meticulously documented over a span of five consecutive days.

### Colony formation assay

HCCC-9810 and RBE cells were harvested and subjected to either EIF3B downregulation, overexpressed EIF3B overexpression, PCNA downregulation, or simultaneous overexpression of EIF3B and downregulation of PCNA. The cells were then trypsinized (Shenggong Biological Engineering Co., Ltd., # T0458-50G), seeded into 6-well plates, and allowed to grow for 5 days to form colonies. Colonies were visualized using a fluorescence microscope (Olympus, Tokyo, Japan) and stained with Giemsa (Dingguo, Shanghai, China) for counting visible clones.

### Cell migration assay

The transwell assay involved culturing cholangiocarcinoma cells transfected with lentivirus in the upper chamber with serum-free medium and transferring the upper chamber to the lower chamber containing 30% FBS. After 72 h, cell staining was carried out using Giemsa, and cell migration ability was quantified.

For the wound-healing assay, cholangiocarcinoma cells transfected with lentivirus were seeded onto a 96-well plate and incubated for 8 and 24 h at 37° C. The migration rate of the cells was then evaluated based on scratch images.

### Flow cytometry assay

10 μL of Annexin V-APC was applied to HCCC-9810 and RBE cells transfected with lentivirus for 10-15 minutes to measure cell apoptosis. The apoptotic cells were then analyzed using FACSCalibur (BD Biosciences, San Jose, CA, USA).

For the double-staining method, lentivirus-transfected cells were cultured in a 6-well plate with 2 mL per well. Subsequently, the cells were washed with D-Hanks (4° C, pH=7.2~7.4) and stained in the dark by adding 5 μL Annexin V-APC and 5 μL propidium iodide (PI). The level of cell apoptosis was assessed using the FACSCalibur flow cytometer (BD Biosciences, San Jose, CA, USA).

### PrimeView human gene expression array

Affymetrix Human GeneChip PrimeView analysis was performed on HCCC-9810 cells transfected lentivirus expressing shEIF3B/shCtrl to assess the statistical significance of the raw data using a Welch t-test with Benjamini-Hochberg false discovery rate (FDR) correction. Significant difference analysis and functional analysis were performed using Ingenuity Pathway Analysis (IPA) was executed, with |Z - score| > 0 considered indicative of significance.

### Bioinformatics analysis

We conducted an analysis on cholangiocarcinoma and normal tissue samples from the TCGA database, with data sourced from GDC (https://portal.gdc.cancer.gov/). RNA sequencing data was utilized, and the standardization and differential analysis were performed using the R package DESeq2 on Count Data.

### The construction of nude mouse tumor formation model

The Ethics Committee of the Third Xiangya Hospital of the Central South University approved all animal experiments, conducted in accordance with the European Parliament Directive (2010/63/EU). A xenograft model was established in four-week-old female BALB-c nude mice by subcutaneously injecting 4×10^6^ RBE cells with shEIF3B and shCtrl. Tumor volume was measured throughout the entire experimental period. On the final day, 0.7% sodium pentobarbital was intraperitoneally injected for several min, followed by fluorescence observation using the *in vivo* imaging system (IVIS Spectrum, Perkin Elmer, Waltham, MA, USA). Subsequently, the mice were euthanized, and their tumors were weighed and photographed. The tumors were then frozen in liquid nitrogen and kept at -80° C.

### Analyses of protein degradation

Protein degradation analysis was conducted on HCCC-9810 cells through treatment with 0.2 mg/mL cycloheximide (CHX). Concurrently, protein ubiquitination of PCNA was examined in EIF1B-depleted HCCC-9810 cells using a ubiquitin antibody. Antibodies used were detailed in Supplementary Table 1.

### Statistical analysis

Statistical analysis was carried out utilizing GraphPad Prism 8 (San Diego, CA, USA) and SPSS 19.0 (IBM, SPSS, Chicago, IL, USA). To evaluate the statistical significance of the data, Student’s t-test and one-way ANOVA were employed. Spearman correlation analysis and Mann-Whitney U analysis were utilized to assess the association between EIF3B expression and pathological characteristics of cholangiocarcinoma patients. *P* < 0.05 was considered statistically significant. All experiments were performed in triplicate.

## Supplementary Material

Supplementary Figures

Supplementary Tables
