# Supplementary material for: EIF3B stabilizes PCNA by counteracting SYVN1-mediated ubiquitination to serve as a promotor in cholangiocarcinoma
Source: Aging (Albany NY). 2024 Apr 29;16(8):7311–30. doi: 10.18632/aging.205759 (PMC11087095; doi:10.18632/aging.205759)
Supplement: Supplementary Figures [file aging-16-205759-s001.pdf]

SUPPLEMENTARY FIGURES

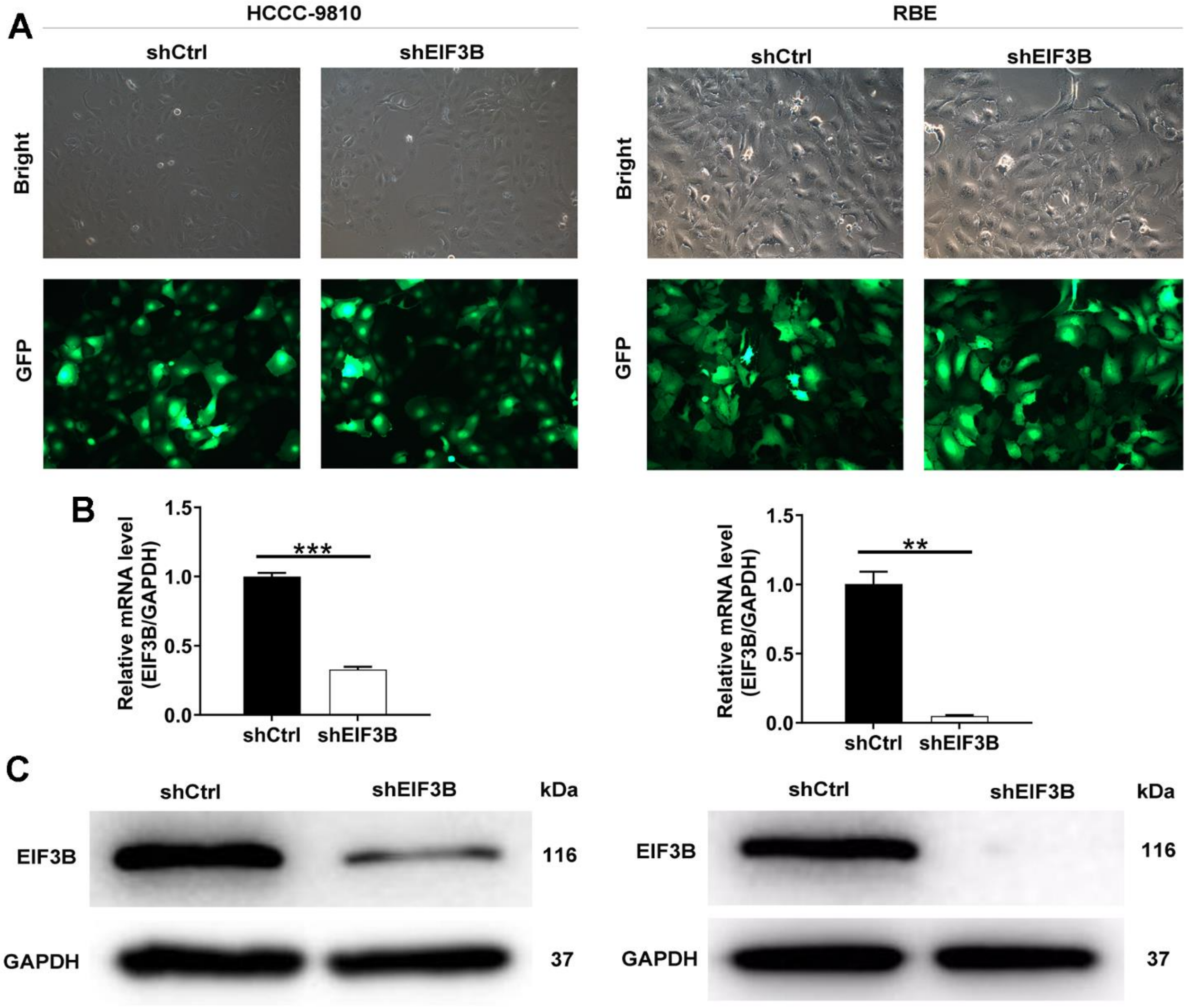

Supplementary Figure 1. (**A**) The transfection efficiencies of shEIF3B in HCCC-9810 and RBE cells were evaluated through observing the fluorescence inside cells. Magnification times: 200 ×. (**B**, **C**) The knockdown efficiency of EIF3B was assessed via qRT-PCR (**B**) and western blot analysis (**C**). The experiments were in triplicate.

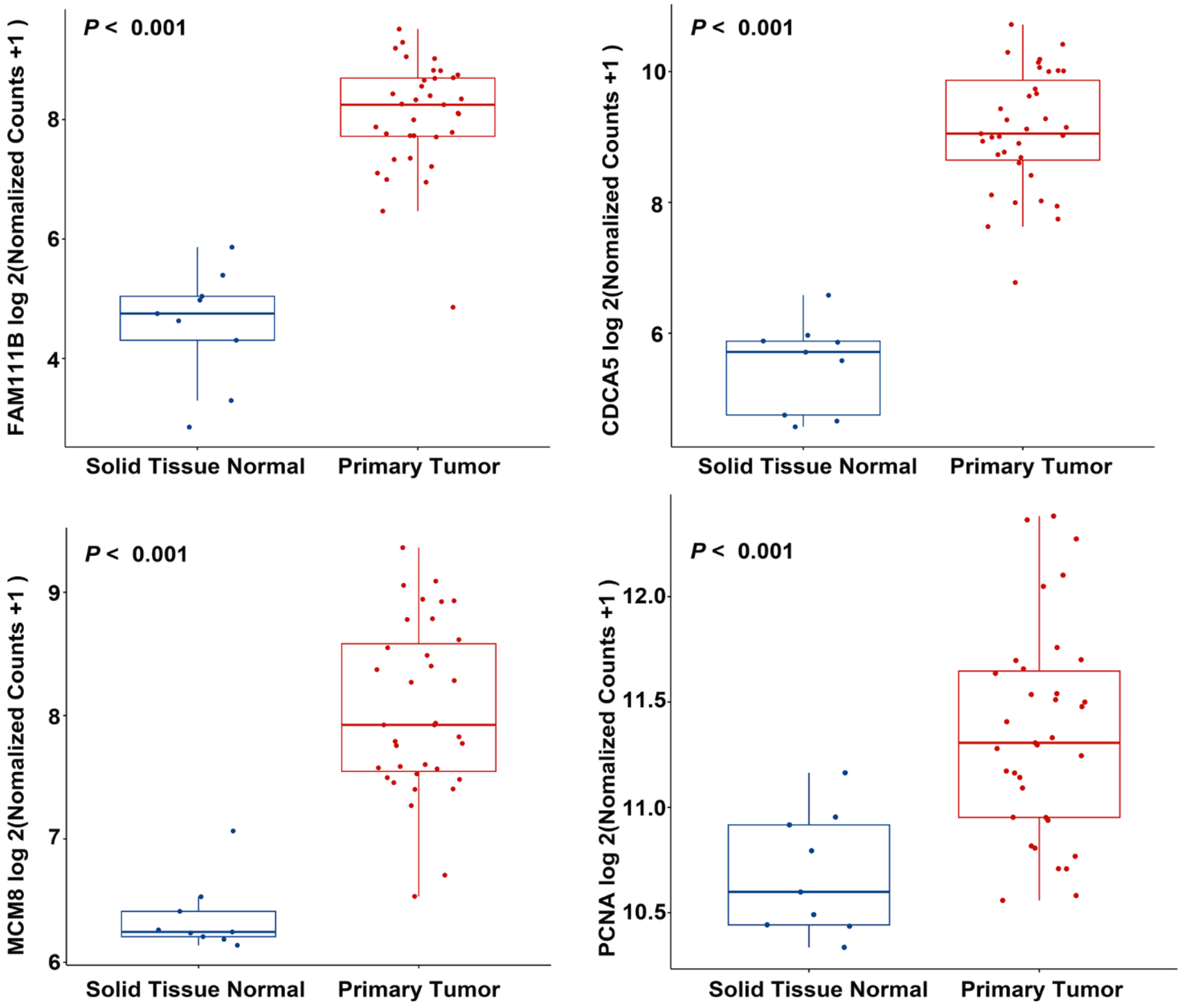

Supplementary Figure 2. Differential expression analysis of CDCA5, FAM111B, MCM8, and PCNA based on cholangiocarcinoma and normal tissues samples from the TCGA database.

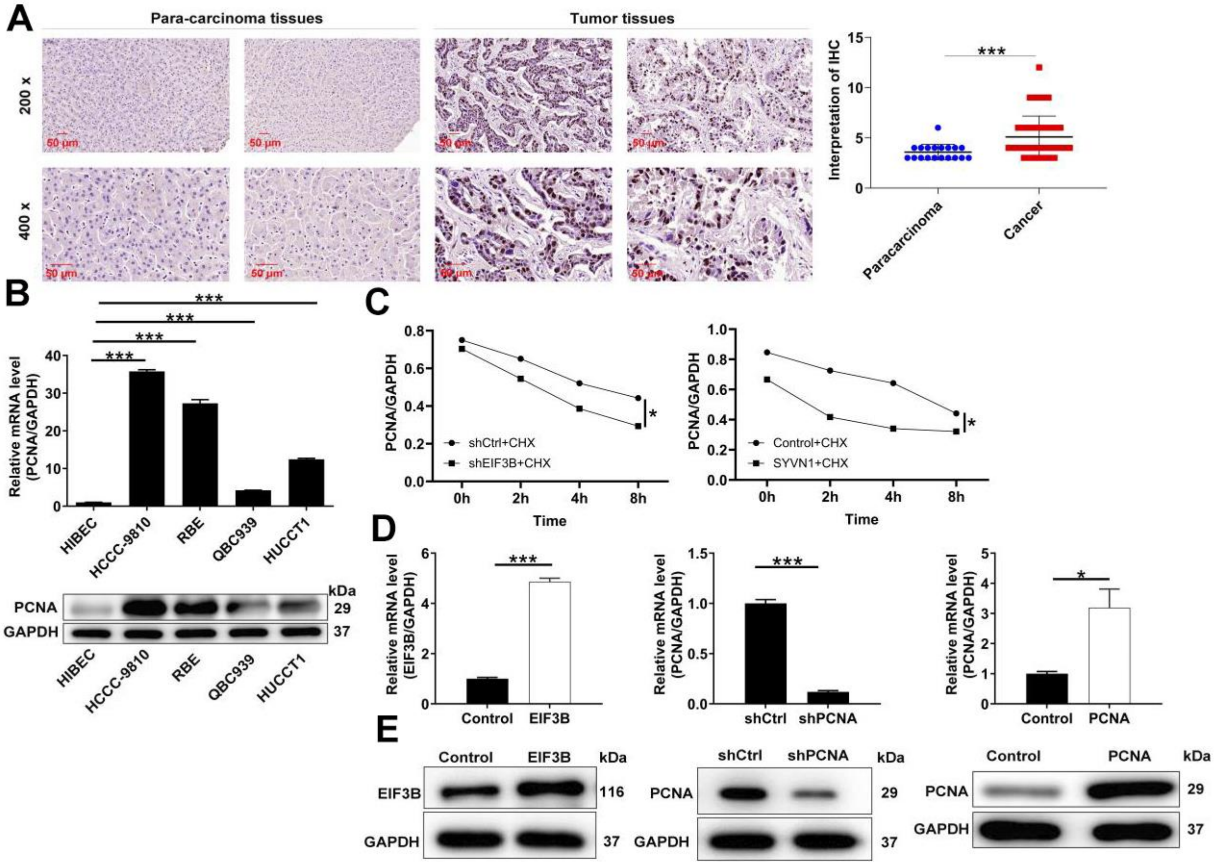

Supplementary Figure 3. (**A**) Typical images and quantitative data illustrations of PCNA immunohistochemical staining in cholangiocarcinoma tissues and para-carcinoma tissues. Scale bar: 50 µm. Magnification times: 200 ×, 400 ×. (**B**) PCNA mRNA and protein levels in cholangiocarcinoma cell lines (HCCC-9810, RBE, HUCCT1, QBC939) and HIBEC cell lines. (**C**) Additional quantitative data supporting the accelerated degradation of PCNA protein in response to EIF3B knockdown or SYVN1 overexpression. (**D**, **E**) Assessment of EIF3B overexpression and PCNA overexpression/knockdown in HCCC-9810 or REB cells by qRT-PCR (**D**) and western blot (**E**). * $P < 0.05$, *** $P < 0.001$.
