# Supplementary material for: EIF3B stabilizes PCNA by counteracting SYVN1-mediated ubiquitination to serve as a promotor in cholangiocarcinoma
Source: Aging (Albany NY). 2024 Apr 29;16(8):7311–30. doi: 10.18632/aging.205759 (PMC11087095; doi:10.18632/aging.205759)
Supplement: Supplementary Tables [file aging-16-205759-s002.pdf]

SUPPLEMENTARY TABLES

Supplementary Table 1. Antibodies used in western blot and IHC.

Primary antibodies	Dilution in WB	Source species	Company	Catalog No.
EIF3B	1:1000	Rabbit	abcam	ab124778
Bcl-2	1:3000	Rabbit	Proteintech	12789-1-AP
Caspase-3	1:2000	Mouse	Proteintech	66470-2-Ig
Survivin	1:2000	Rabbit	novus	10508-1-AP
CDCA5	1:1000	Rabbit	abcam	ab192237
FAM111B	1:1000	Rabbit	thermo	PA5-28529
MCM8	1:1000	Rabbit	abcam	ab191914
PCNA	1:1000	Mouse	abcam	ab29
P21	1:750	Rabbit	Proteintech	10355-1-AP
SYVN1	1:50/1:2000	Rabbit	Proteintech	13473-1-AP
Ubiquitin	1:1000	Mouse	Santa Cruz	sc-47721
GAPDH	1:3000	Rabbit	Bioworld	AP0063
DYKDDDDK Tag	1:50/1:1000	Rabbit	CST	14793
Secondary antibody	Dilution		Company	Catalog No.
Goat Anti-Rabbit	1:3000		Beyotime	A0208
Goat Anti- Mouse	1:3000		Beyotime	A0216
**Primary antibodies**	**Dilution in IHC**	**Source species**	**Company**	**Catalog No.**
EIF3B	1:50	Rabbit	abcam	**ab124778**
PCNA	1:100	Rabbit	Proteintech	10205-2-AP
Ki67	1:200	Rabbit	abcam	Ab16667
Secondary antibody	Dilution		Company	Catalog No.
Goat Anti-Rabbit	1:400		abcam	A6721

Supplementary Table 2. Primers used in qRT-PCR.

Gene	Forward primer sequence (5′-3′)	Reverse primer sequence (5′-3′)
EIF3B	CCTGAAGAGGATGGGAAGACA	AAGAGGTTGACCCGGAATG
FANCA	GTTTGAGCATACGGGGAACA	TGGCAGTAGGTGGAGTACAGAG
IPO5	GCCATCACTGAAGCACATCG	TTCCCAACAGCCAGACCAAT
BARD1	ATCCTCAGTAGAAAGCCCAAGC	TCAGAATCGGGTCTCGCATG
LIG1	ATGGTGGAGACGCTGAGCAA	TGGTTGAGGCTGAGGTAGAGGA
MCM8	ATGGCTTTTCTTTGTGCTGC	CCAGTCCATCGTAACTGTGAGA
MDM2	GGTGAGGAGCAGGCAAATGT	CGAAGCTGGAATCTGTGAGGT
HSDL2	AGTCCACCACTGAACCTAAATCC	TCTGCCATTCCAAGCACATAC
BRCA1	TGGCAACATACCATCTTCAACC	TGTCAATTCTGGCTTCTCCCT
CDCA5	CCGAGCATCCTCCCTGAAAT	CATGGGCCACGATCCTCTTTA
ERCC6L	AAAAGTCAAGCAACCCAGAGG	GTAAAGGCACAAGTCGTATCCA
CCNA2	AGCCTGCGTTCACCATTCA	GGGCATCTTCACGCTCTATTTT
PCNA	GGCGTGAACCTCACCAGTATGT	CGTTATCTTCGGCCCTTAGTGTA
CCNB1	AAACTTTGGTCTGGGTCGGC	TGCTGCAATTTGAGAAGGAGG
CHAF1A	CAGTGATGTCGTCATCGTGG	TGAGTGCCGTCTTCTTATTCC
DDB2	CAGGACCCTCCACCAGCATAA	GCCACGCCAAGGATGTAGCC
RPA2	ATTGTGGGGATCATCAGACATG	CAGTGTTTTCACTGCTGGTGTC
SKP2	ATAGAAGTGTCCACCCTCCACG	CACCCAGAAAGGTTAAGTCGC
TMPO	CCCCTATGAAGCATCTACACCA	GCCAAGGGAACATACTTAGGAAC
FAM111B	CCAGACAATTCCCAGGATTAGA	TAGCATACCGCCTACCCAGA
GAPDH	TGACTTCAACAGCGACACCCA	CACCCTGTTGCTGTAGCCAAA
